# Effects of tiotropium on the risk of coronary heart disease in patients with COPD: a nationwide cohort study

**DOI:** 10.1038/s41598-022-21038-1

**Published:** 2022-10-05

**Authors:** Jiyoung Shin, Jin Hwa Lee

**Affiliations:** 1 Department of Health Care Policy Research, Korea Institute for Health and Social Affairs, Sejong, Republic of Korea; 2 Division of Pulmonary and Critical Care Medicine, Department of Internal Medicine, College of Medicine, Ewha Womans University, 25 Magokdong-ro 2-gil Gangseo-gu, Seoul, 07804 Republic of Korea

**Keywords:** Chronic obstructive pulmonary disease, Cardiovascular diseases

## Abstract

Inhaled long-acting muscarinic antagonist (LAMA) is recommended for the treatment of chronic obstructive pulmonary disease (COPD). However, there is still concern that LAMA may cause cardiovascular adverse events in COPD patients. Therefore, this study aimed to determine whether the administration of tiotropium, the first commercially available LAMA, could increase the risk of coronary heart disease (CHD) in COPD patients through a nationwide cohort study. We used the Korean National Health Insurance Service-National Sample Cohort (NHIS-NSC) database between 2002 and 2014 for the analysis. We applied a washout period of COPD diagnosis during 2002–2003 and excluded the patients who used an inhaler before the diagnosis of COPD. We also excluded patients who were diagnosed with CHD before inhaler use. Among a total of 5787 COPD patients, 1074 patients were diagnosed with CHD. In the Cox regression models with time-dependent tiotropium usage, we found that tiotropium significantly increased the risk of CHD in a subgroup of age $\ge \hspace{0.17em}$55 years compared to non-users of tiotropium (adjusted hazard ratio [aHR], 1.24; 95% confidence interval [CI], 1.003–1.54). When analyzed by dividing into tertiles (high/middle/low) according to the cumulative tiotropium exposure, the high tertile exposure group of tiotropium was associated with a higher risk of CHD compared with the low tertile exposure group of tiotropium. Additionally, the risk of CHD was higher in the high tertile exposure group of tiotropium in the age 55 and older group and in the never smoker group. When prescribing tiotropium for COPD patients, particularly those over 55 years of age and never-smokers, it is desirable to evaluate the risk of CHD in advance and closely follow-up for CHD occurrence.

## Introduction

Chronic obstructive pulmonary disease (COPD) is a common lung disease characterized by persistent airflow limitations and chronic respiratory symptoms. COPD can be caused by environmental factors such as smoking in a genetically susceptible population^1^. In COPD patients, pharmacological treatment is conducted to alleviate respiratory symptoms and prevent acute exacerbations, one of the main prognostic factors for COPD^2^. As a first-line therapy, based on the patient’s clinical status, inhalation medication including long-acting muscarinic antagonists (LAMA), long-acting beta-2 agonists (LABA), and/or inhaled corticosteroids (ICS) are currently used^3^.

Coronary heart disease (CHD) is a common comorbidity of COPD and a leading cause of COPD mortality^4,5^. Some inhalers, including ICS, have been shown to have a protective effect on CHD^6,7^. On the other hand, some researchers have shown that the use of LAMA may increase the cardiovascular risk^8–10^. In a meta-analysis of clinical trials, tiotropium Respimat was significantly associated with cardiovascular mortality compared to placebo^11^. In the largest Respimat study, they identified an increased risk of cardiovascular mortality in COPD patients with known rhythm and cardiac disorders who were prescribed Respimat. However, there was no significant increased risk of myocardial infarction when tiotropium handihaler was administered^12^. However, this analysis included data from the understanding potential long-term impacts on function with tiotropium (UPLIFT) trial, which did not monitor adverse events for more than 40% of patients who already terminated the treatment ^13^.

In Korea, the National Health Insurance Service (NHIS) operates the national sample cohort data, which includes a million representative subjects. Using this data, it is possible to evaluate a disease incidence and medication usage or risk factors in a large-scale national population. The goal of this study was to evaluate the effect of tiotropium, the first commercially available LAMA, on CHD in COPD patients using national population-based data.

## Methods

### Data source

The Korea national health insurance service (NHIS), a health insurance system with universal coverage, constructed the national health information database (NHID) based on the claim data from 2000, from almost the entire Korean population (> 97%)^14^. This database includes demographic and other medical claims data of the Korean population. In 2015, the NHIS constructed the NHIS-National Sample Cohort (NSC) population-based data, which has a follow-up period between 2002 and 2013, consisting of ~ 1 million individuals^14^. In 2017, NHIS released a second version of the NHIS-NSC population-based cohort data, which has a follow-up period between 2002 and 2015. In this cohort, about 1 million subjects were extracted based on systematic, stratified, random sampling method with 2142 strata (constructed using age groups, sex, residential area and income level) from the target population (48,222,537 individuals)^15^. The present study used this second version of NHIS-NSC, which contains information about the participant’s insurance eligibility, medical treatments, medical care institutions, and general health examinations. General health examinations comprise information from nationwide health examinations conducted by the NHIS in 2002–2015 and it shows the health examination results along with the subject’s lifestyle and health behavior, which was obtained by questionnaire.

### Study population and case definition

In this study, we used NHIS-NSC between 2002 and 2014. We excluded the year 2015 because the first usage of LAMA/LABA medication occurred in 2015 and this study only focused on the effect of LAMA medication before the LAMA/LABA prescription started. COPD patients were defined as those who were diagnosed with COPD at least twice a year based on the Korean Classification of Diseases, 6th revision (KCD-6). KCD-6 is a modified version of the International Classification of Disease, 10th revision (ICD-10). The KCD-6 code criteria for COPD include J42.x to J44.x except J430 (Supplementary Table 1). We included only patients who were prescribed their first inhaler after being diagnosed with COPD. In addition, a total of two years between 2002 and 2003 was set as a washout period, and patients diagnosed with COPD or prescribed inhalers during this period were excluded.

To define our main outcome, CHD, we used KCD-6 code and procedure code, which could be utilized in the NHIS-NSC medical treatment database. In accordance with the American Heart Association, the KCD-6 code between I20 and I25 was used to extract the CHD patients (Supplement Tables 1, 2)^16^. After considering the procedure code, a CHD event was defined as a case in which one or more of the following three conditions were satisfied: (i) At least one hospitalization based on the KCD-6 code for CHD; (ii) At least two outpatient visits based on the KCD-6 code for CHD; (iii) At least one revascularization based on the procedure code defined as coronary artery bypass grafting (‘O1641’, ‘O1642’, ‘O1647’, ‘OA641’, ‘OA642’, ‘OA647’) or percutaneous coronary intervention (‘M6551’, ‘M6552’, ‘M6561-4’, ‘M6571’ and ‘M6572’)^17–19^. Patients who were diagnosed with CHD before using inhalers were excluded.

### Exposure to LAMA and non-LAMA inhaler

We defined an inhaler user as a patient who was prescribed inhalation respiratory medicine at least two times in a year between January 1, 2004 and December 31, 2014. We set this twice prescription criteria to exclude the possible miscoding of prescription data. LAMA (tiotropium); long-acting beta-2 agonists (LABA; formoterol or salmeterol); inhaled corticosteroids (ICS; beclomethasone, budesonide, ciclesonide, flunisolide, fluticasone, or triamcinolone); a combination of ICS/LABA (budesonide/formoterol, fluticasone/salmeterol), short-acting beta-2 agonists (SABA; fenoterol, procaterol, salbutamol, or terbutaline); short-acting muscarinic antagonists (SAMA; ipratropium); and a combination of SABA/SAMA (ipratropium/salbutamol) were included as an inhaled respiratory medicine. LAMA users were defined as subjects who were prescribed tiotropium at least twice in a year after a COPD diagnosis, and non-LAMA users were defined as subjects who did not meet the criteria of LAMA users.

The index date from which the follow-up period began was identified as the point at which the first inhaler of the inhaler users was prescribed. The time before the use of LAMA was reallocated to non-LAMA user to overcome the immortal time bias. All the subjects are followed-up to the earliest date of CHD onset, death or the last day of 2014. LAMA cumulative dose was calculated by aggregating the total dose of tiotropium medication prescribed during a specific patient follow-up period. The year of the first inhaled respiratory medicine prescription was considered an inhaler initiation year.

### Statistical analysis

Categorical characteristics between CHD and non-CHD groups were compared by using χ^2^ test, and continuous characteristics were compared by using *t *test. The Kaplan–Meier curves were established based on LAMA use, sex, cumulative dose of LAMA (low, < 2544.3 µg of tiotropium; high, $\ge \hspace{0.17em}$2544.3 µg of tiotropium) and smoking status, with the significance based on the log-rank test. A time-dependent Cox proportional hazards model was used to estimate the association between LAMA usage and CHD onset in COPD patients, and adjusted hazard ratio (aHR) and 95% confidence interval (CI) were calculated. The cumulative exposure to LAMA was classified into tertiles again (low, < 112.5 µg of tiotropium; middle, 112.5–2544.3 µg of tiotropium; high, $\ge \hspace{0.17em}$2544.3 µg of tiotropium), and the CHD risk was compared based on the tertile with a low cumulative exposure to LAMA. In this analysis, the cumulative exposure of non-LAMA users and LAMA users prior to the first LAMA prescription was considered zero.

For the covariates, we used demographic characteristics including age, sex, body mass index (BMI) and household income level (4 strata). We also included smoking status^20^ and Charlson comorbidity index (CCI)^21^ as covariates. All covariates were considered CHD risk factors and these variables were assessed from the latest record of the national health examination from the year of CHD diagnosis. For the non-CHD patients, the information was collected from the latest record of national health examination.

We also performed subgroup analysis based on patient’s age and sex, which are patients aged $\ge \hspace{0.17em}$55, men < 55, and women < 55. We used this cutoff criterion because estrogen may have a cardioprotective effect and lower the risk of CHD of premenopausal women compared to the men^22^. After menopause, there is no known difference in the risk of CHD between men and women^23^. Considering that 97.6% of Korean women experience menopause before 55, the study subjects were divided based on the age of 55 years^24^. We also tested interaction term between the LAMA use and the subgroup variables in the Cox models. For the sensitivity analysis, we performed the model excluding the patients who are aged between 30 and 39. All analyses were conducted using SAS version 9.4 software (SAS Institute, Cary, NC, USA) and R studio 1.0.136 (R studio, Inc). All *p *values were two-sided, and those < 0.05 were considered to indicate statistical significance.

### Ethics approval

All methods were performed in accordance with the relevant guidelines and were approved by the Institutional Review Board of Ewha Womans University Hospital, Seoul, Republic of Korea (IRB number: SEUMC 2021-03-006). Informed consent from participants was waived by IRB because NHIS-NSC does not contain any identifying information and the study involved minimal risk to human subjects.

## Results

### Subject enrollment

**Figure 1:**
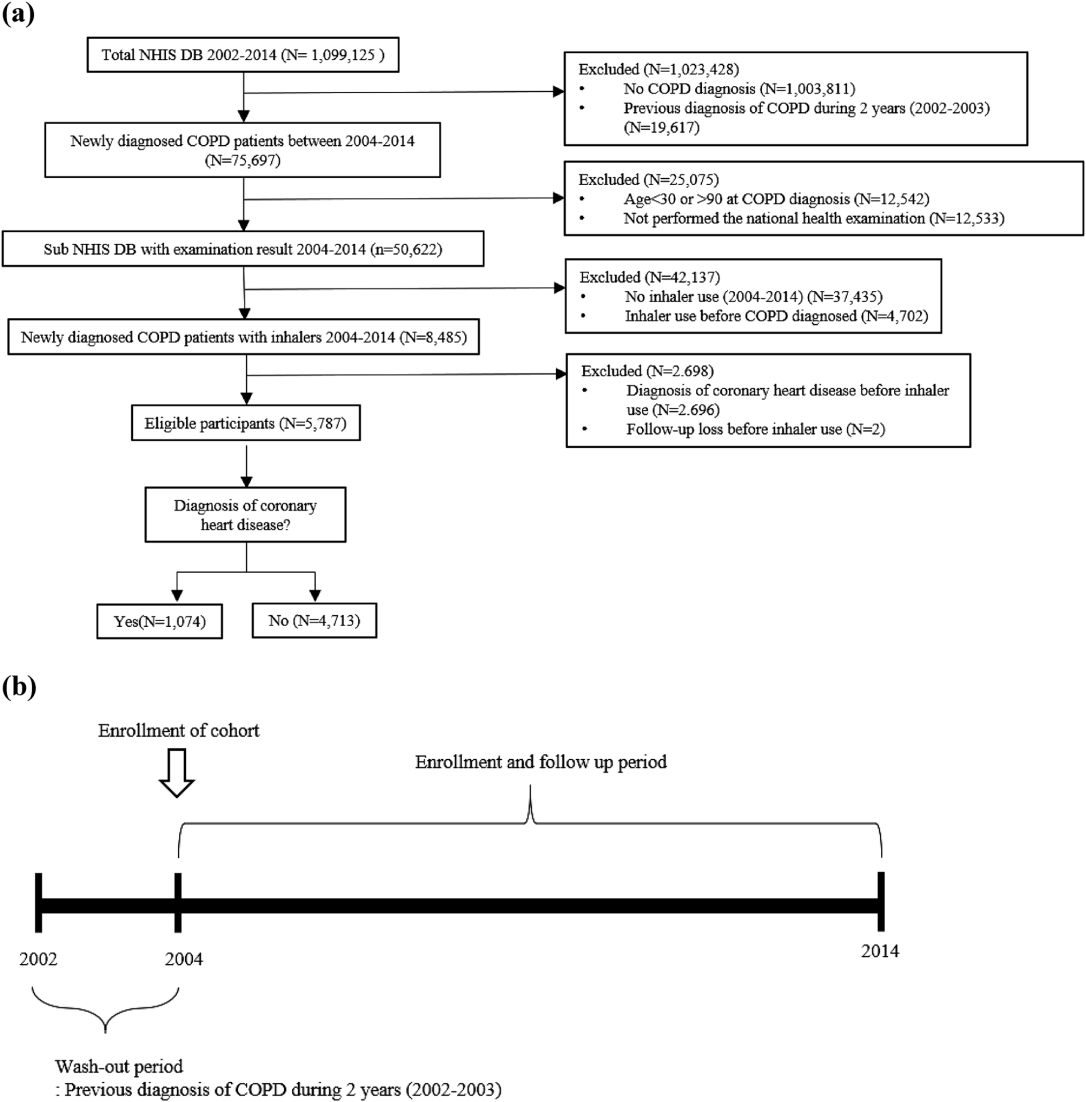
Study design (**a**) Flow diagram of the study design (**b**) Study design over time. NHIS DB, National Health Insurance Service database; COPD, chronic obstructive pulmonary disease.

Subjects diagnosed with COPD between 2002 and 2014 were selected from the NHIS-NSC. Among them, patients diagnosed with COPD for 2 years from 2002 to 2003 (wash-out periods) were excluded to include only those with a first diagnosis of COPD in this study. For the remaining 75,697 COPD patients, age criteria (30–90 years old) were applied, and patients without national health examination information were excluded. After that, patients with no history of inhaler use were excluded, and patients who used an inhaler before a COPD diagnosis were excluded. After that, out of 8485 COPD patients, 5787 patients were finally selected for this study, excluding those who were diagnosed with CHD before inhaler use or were censored prior to inhaler use (Fig. 1).

### Baseline demographics

Of the 5787 COPD patients, 1074 (18.6%) were diagnosed with CHD. The mean follow-up period was 3.6 years, and the mean age was 61.4 years. In the CHD group, the proportion of patients over 55 years of age was 83.3%, which was significantly higher than that of the non-CHD group. Males accounted for 56.8% of all patients, and the proportion of females was significantly higher in non-CHD group (43.9%) than in CHD group (40.3%). The mean CCI in CHD patients was 3.1 and the mean CCI in non-CHD patients was 2.0. There was no difference in the LAMA prescription rates between the CHD group and the non-CHD group, 24.1% and 22.8%, respectively. In addition, the presence or absence of asthma in each patient’s inhaler initiation year was evaluated, and this was identified based on the KCD-6 codes (J45, J46) at least twice in the inhaler initiation year (Table 1).

**Table 1:** Characteristics of the study subjects^a^.

Variables	Total (n = 5,787)	CHD (n = 1,074)	Non-CHD (n = 4,713)	*p *value^b^
Follow up duration (years)	3.6 ± 2.9	2.5 ± 2.4	3.9 ± 2.9	< 0.001
	2.9 [1.1, 5.7]	1.8 [0.6, 3.9]	3.2 [1.3, 6.0]	
**Age (years)**	61.4 ± 12.7	65.4 ± 10.7	60.5 ± 12.9	< 0.001
< 55	1655 (28.6%)	179 (16.7%)	1476 (31.3%)	< 0.001
≥ 55	4132 (71.4%)	895 (83.3%)	3237 (68.7%)	
**Sex**				
Men	3285 (56.8%)	641 (59.7%)	2644 (56.1%)	0.03
Female	2502 (43.2%)	433 (40.3%)	2069 (43.9%)	
**Smoking status**				
Never smokers	3378 (58.4%)	634 (59.0%)	2744 (58.2%)	0.49
Former smokers	1020 (17.6%)	176 (16.4%)	844 (17.9%)	
Current smokers	1389 (24.0%)	264 (24.6%)	1125 (23.9%)	
**Body mass index (kg/m**^**2**^**)**	23.3 ± 3.7	23.4 ± 3.9	23.3 ± 3.6	0.49
**CCI**	2.2 ± 1.8	3.1 ± 2.0	2.0 ± 1.7	< 0.001
**Household income levels**				
0–20%	894 (15.5%)	171 (15.9%)	723 (15.3%)	0.37
20–40%	775 (13.4%)	139 (12.9%)	636 (13.5%)	
40–60%	991 (17.1%)	179 (16.7%)	812 (17.2%)	
60–80%	1241 (21.5%)	214 (19.9%)	1027 (21.8%)	
80–100%	1540 (26.6%)	294 (27.4%)	1246 (26.4%)	
**Medication**				
LAMA	1333 (23.0%)	259 (24.1%)	1074 (22.8%)	0.35
ICS	2433 (42.0%)	511 (47.6%)	1922 (40.8%)	< 0.001
ICS/LABA	2337 (40.4%)	470 (43.8%)	1867 (39.6%)	< 0.001
LABA	156 (2.7%)	16 (1.5%)	140 (3.0%)	0.01
SABA	4347 (75.1%)	819 (76.3%)	3528 (74.9%)	< 0.001
SABA/SAMA	103 (1.8%)	37 (3.5%)	66 (1.4%)	< 0.001
SAMA	2085 (36.0%)	436 (40.6%)	1649 (35.0%)	< 0.001
**Asthma status**^**c**^				
No	850 (14.7%)	123 (11.5%)	727 (15.4%)	0.001
Yes	4937 (85.3%)	951 (88.6%)	3986 (84.6%)	

CHD, coronary heart disease; CCI, Charlson comorbidity index; ICS, inhaled corticosteroids; LABA, long-acting beta-2 agonists; LAMA, long-acting muscarinic antagonists; SABA, short-acting beta-2 agonists; SAMA, short-acting muscarinic antagonists.

^a^ Data are n (%), or mean ± SD, or median [interquartile range].

^b^ *p* value for chi square test or *t* test.

^c^ In the year of inhaler initiation.

### Incidence of CHD

**Figure 2:**
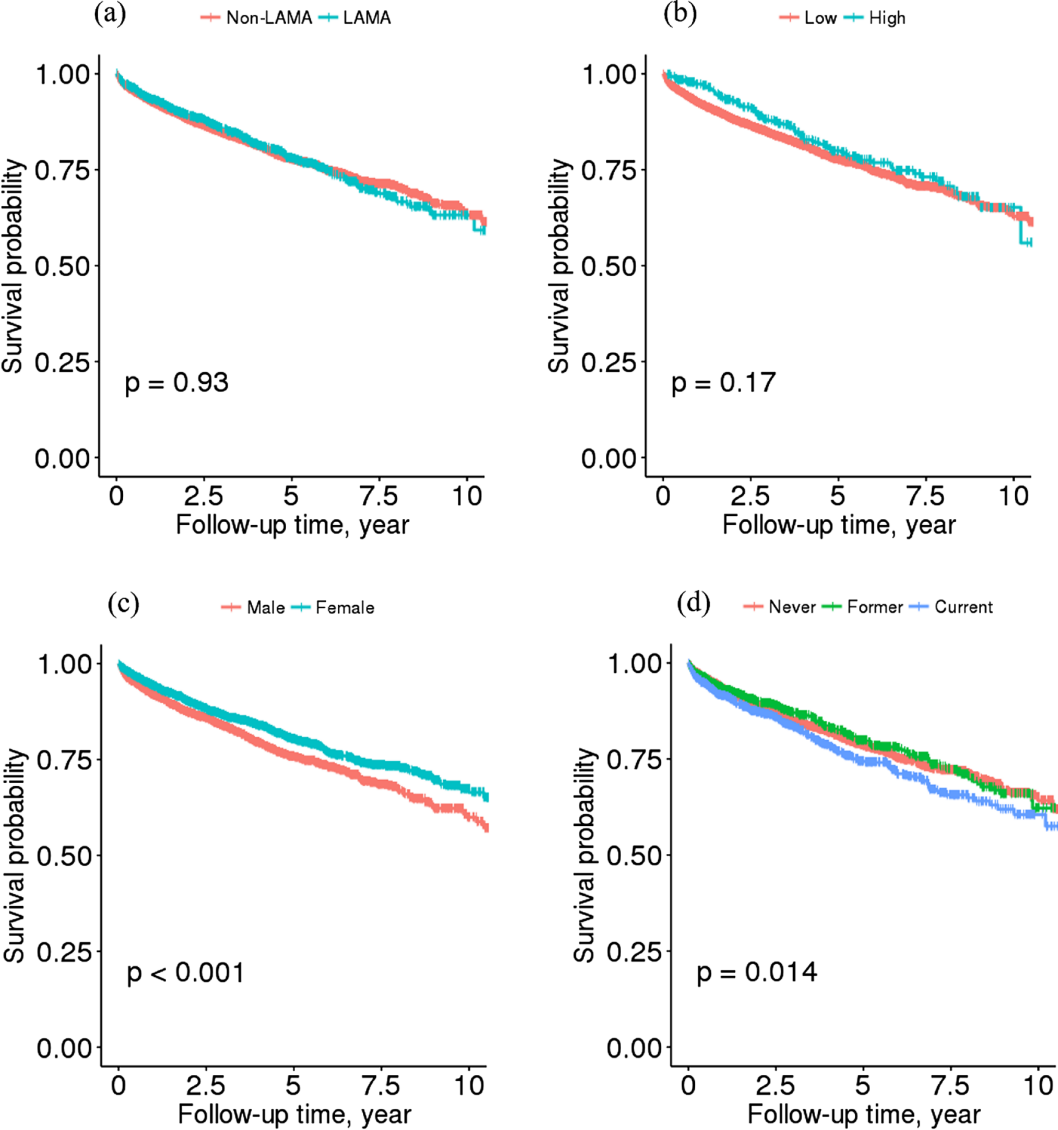
Kaplan Meier curves according to (**a**) use of LAMA, (**b**) cumulative dose of LAMA^a^, (**c**) sex, and (**d**) smoking status among COPD patients with inhaler use. ^a^ High cumulative group is the highest tertile group of cumulative dose distribution of tiotropium (High tertile of cumulative dose is $\ge \hspace{0.17em}$2544.3 $\mathrm{\mu g}$ of tiotropium, Low tertile of cumulative dose is < 2544.3 µg of tiotropium).

The Kaplan–Meier (KM) curves according to use of LAMA (*P* = 0.93, Fig. 2a) and cumulative dose of LAMA (*P* = 0.17, Fig. 2b) suggested the proportional hazards assumption was not fulfilled. On the other hand, KM curves according to sex (*P* < 0.001, Fig. 2c) and smoking status (*P* = 0.014, Fig. 2d) showed significant differences in CHD occurrence.

### LAMA usage and the risk of CHD

The crude models showed that LAMA use was associated with a higher risk of CHD in the overall population and several subgroups. However, in the adjusted models, only the subgroup 55 years and older showed an association between LAMA use and an increased risk of CHD (HR, 1.24; 95% CI, 1.003–1.54) (Table 2).

**Table 2:** Association between tiotropium administration and the risk of coronary heart disease in COPD patients ^a^.

Subgroup	No. of CHD cases/ Total No. of subgroup patients	Crude hazard ratio (95% CI)	*p* value	Adjusted hazard ratio (95% CI)	*p* value
Total^b^	1074/5787	1.58 (1.29–1.93)	< 0.001	1.23 (1.00–1.51)	0.052
Adults ≥ 55 years^b^	895/4132	1.41 (1.14–1.74)	0.002	1.24 (1.003–1.54)	0.047
Males < 55 years^c^	75/700	1.97 (0.89–4.35)	0.09	1.70 (0.76–3.82)	0.20
Females < 55 years^c^	104/955	0.40 (0.06–2.86)	0.36	0.37 (0.05–2.71)	0.33
Never smoker^d^	634/3378	1.64 (1.22–2.21)	0.001	1.15 (0.85–1.55)	0.38
Former smoker^d^	176/1020	1.40 (0.93–2.13)	0.11	1.30 (0.85–1.98)	0.22
Current smoker^d^	264/1389	1.65 (1.13–2.40)	0.009	1.33 (0.91–1.95)	0.14

COPD, chronic obstructive pulmonary disease; CI, confidence interval.

^a^ Tiotropium LAMA usage is analyzed as time-dependent covariates.

^b^ Adjusted HRs were adjusted for age, sex, body mass index, household income level, Charlson comorbidity index, and smoking status.

^c^ Adjusted HRs were adjusted for age, body mass index, household income level, Charlson comorbidity index, and smoking status.

^d^ Adjusted HRs were adjusted for age, sex, body mass index, household income level, and Charlson comorbidity index.

In the adjusted model of the association between cumulative dose of LAMA exposure and the risk of CHD in COPD patients, the overall population, 55 years of age and older, and never-smoker group were found to have an increased risk of CHD with increasing cumulative LAMA dose (Table 3).

**Table 3:** Association between cumulative dose of tiotropium exposure and the risk of coronary heart disease in COPD patients^a^.

Subgroup	No. of CHD cases/ Total No. of subgroup patients	Crude hazard ratio (95% CI)	*p* value	Adjusted hazard ratio (95% CI)	*p* value
Total^b^	1074/5787	1.02 (1.01–1.03)	< 0.001	1.01 (1.002–1.022)	0.020
Adults ≥ 55 years^b^	895/4132	1.02 (1.01–1.03)	0.002	1.01 (1.002–1.022)	0.015
Males < 55 years^c^	75/700	1.01 (0.95–1.08)	0.73	1.01 (0.94–1.08)	0.88
Females < 55 years^c^	104/955	1.03 (0.96–1.11)	0.35	0.98 (0.91–1.06)	0.63
Never smoker^d^	634/3378	1.04 (1.03–1.05)	< 0.001	1.03 (1.02–1.04)	< 0.001
Former smoker^d^	176/1020	1.00 (0.96–1.03)	0.73	1.00 (0.96–1.03)	0.79
Current smoker^d^	264/1389	1.01 (0.99–1.04)	0.39	1.00 (0.97–1.02)	0.78

COPD, chronic obstructive pulmonary disease; CI, confidence interval.

^a^ Tiotropium usage is analyzed as time-dependent covariates and converted to mg.

^b^ Adjusted HRs were adjusted for age, sex, body mass index, household income level, Charlson comorbidity index, and smoking status.

^c^ Adjusted HRs were adjusted for age, body mass index, household income level, Charlson comorbidity index, and smoking status.

^d^ Adjusted HRs were adjusted for age, sex, body mass index, household income level, and Charlson comorbidity index.

**Figure 3:**
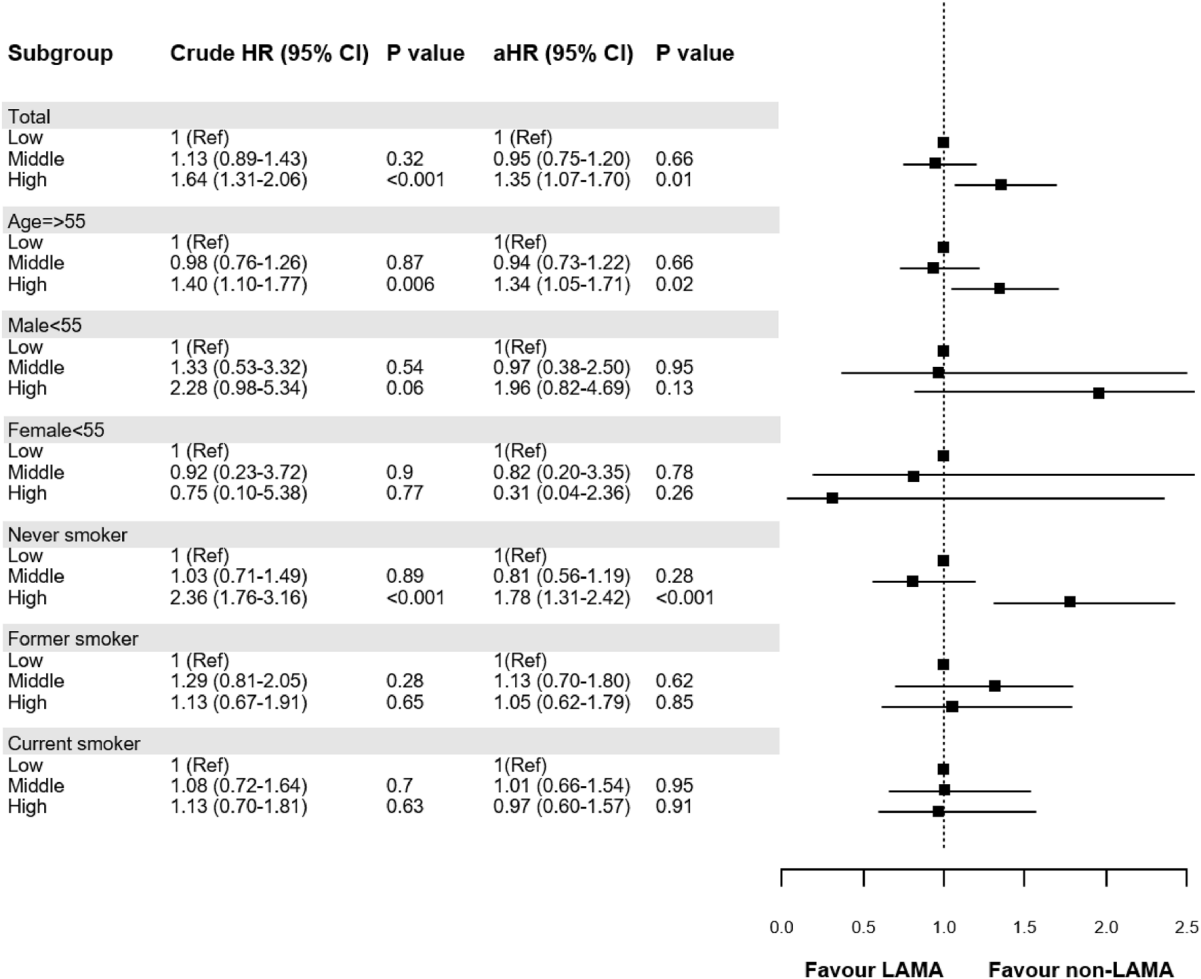
Association between tertiles of tiotropium exposure and the risk of coronary heart disease^a,b^. aHR, adjusted hazard ratio; ^a^ Models adjusted for age, sex, body mass index, household income level, Charlson comorbidity index, and smoking status. ^b^ Low (1st tertile, < 112.5 µg of tiotropium), middle (2nd tertile, 112.5–2544.3 µg of tiotropium), and high (3rd tertile, $\ge$ 2544.3 µg of tiotropium).

When the cumulative dose of LAMA was divided by tertiles, the higher tertile of the cumulative dose of LAMA showed a higher risk of CHD in the overall population, the age group 55 years and older, and the never-smoker group (Fig. 3).

When we evaluated the differential effect between the subgroups by adding interaction term in the model, we did not find an interaction between LAMA and age group. However, interaction term between LAMA and sex, and interaction term between LAMA and smoking status had a *p *value < 0.1 in some models (Supplementary Table 3, 4). In the sensitivity analysis including patients aged 40 and over, the results were almost similar to the Tables 2 and 3 (Supplementary Table 5, 6).

## Discussion

Using the National Health Insurance cohort, we found that tiotropium use was associated with an increased risk of CHD in COPD patients 55 years of age and older who had no history of CHD and were using inhalers. In the model examining the association between COPD patients with or without the use of tiotropium and the risk of CHD, it could not take into account the effect of high exposure to tiotropium because the time after the subject who prescribed a small amount of tiotropium was also considered as a LAMA use. Therefore, we checked for an association between the cumulative dose of LAMA exposure and the risk of CHD, and found a significant increase in the risk of CHD not only for those over 55 years of age, but also for the overall population and never-smoker group. A similar pattern was observed when examining the association between the tertiles of LAMA exposure and the risk of CHD, which showed that a high cumulative dose of LAMA was associated with significantly increased risk of CHD when compared to a low cumulative dose of LAMA in the overall, age $\ge \hspace{0.17em}$55, and never smoker group.

Many studies have investigated the association between LAMA and CHD, but the results are still controversial. In a nested case–control study conducted using the Taiwan National Health Insurance Research Database for medical claims from 2007 to 2011, new initiation of LABA or LAMA in COPD patients was associated with an approximately 1.5-fold increase in serious cardiovascular risk (95% CI, 1.35–1.67 for LABA, 95% CI, 1.28–1.80 for LAMA)^8^. Another study using a meta-analysis also found that tiotripium Soft Mist Inhaler was associated with cardiovascular death in patients with severe COPD (OR 1.96; 95% CI, 1.07–3.60)^25^. On the other hand, in a meta-analysis that combined studies comparing LAMA and placebo in COPD patients to evaluate the cardiovascular safety of LAMA, there was no difference in the incidence of cardiovascular events between LAMA and placebo groups (RR 0.98, 95% CI 0.88–1.09)^26^. While this previous study was based on a meta-analysis about LAMA use versus placebo group, considering that the current study only included participants who used inhalers at least twice a year after COPD diagnosis, our results could implicate that LAMA has an adverse effect compared to other inhalers.

In our study, the cumulative dose of tiotropium was significantly associated with an increased risk of CHD. Unlike previous studies, we used 11 year cohort data and included only the patients newly prescribed tiotropium. We believe that this method may be more helpful in evaluating the effect of tiotropium alone than the methods used in other studies. We also investigated the incidence of CHD only in COPD patients who were not previously diagnosed with CHD. Finally, the effectiveness of tiotropium was evaluated using a time-dependent Cox proportional hazards model to eliminate an immortal time bias that could create the illusion of a treatment effect^27^.

The exact mechanism by which LAMA may have detrimental effects on the heart is still unclear, but there are several proposed studies that may explain this mechanism. Meng-Ting et al. mentioned that the use of LABA and LAMA in COPD may increase inflammatory cytokine levels, and that increased IL-8 may be associated with an increased risk of cardiovascular diseases^8,28,29^. In the previous study, IL-8 is related to the firm adhesion of the rolling monocytes in the early stages of atherogenesis and this interleukin could be involved to more complicated stages of atherosclerosis as well by potentiating plaque angiogenesis^30^. Researchers have also found that LAMA has some affinity for M1 and M2 receptors located outside the airways, suggesting that it may have some association with cardiovascular effects ^31,32^.

In our study, the 55 and older group consistently showed an increased risk of CHD with tiotropium administration in three different models. The older group is known to have a higher risk of CHD than the younger group, and adverse drug reactions may be more pervasive in the older group^33,34^. Our study showed that the elderly was more susceptible to cardiovascular adverse effects of tiotropium.

This study also showed a relationship between tiotropium and CHD in the never-smoker COPD group. Although the mechanism is not clear, one study found that inhaler lung deposition was higher in non-smokers than in smokers^35,36^. This high lung deposition is also likely to increase the positive effect of treatment, but also leads to increased adverse effects of cardiovascular events.

The strength of our study was that the two-year washout period allowed us to reveal the real-world relationship between the cumulative exposure to tiotropium and the occurrence of CHD because only patients who were first diagnosed with COPD, who were prescribed inhaled drugs for the first time, and who had no history of CHD were studied. Previous studies, including randomized controlled trials, were limited in showing an association between LAMA and CHD occurrence because they included COPD patients who were already taking inhaled drugs or had a history of CHD^13^.

Our study has some limitations as well. First, since our study is an observational study, there are some potential confounders, including lung function and underlying diseases, that may contribute to the occurrence of CHD. However, our analysis adjusted for covariates, including age, sex and smoking status, which are the major risk factors for CHD. In addition, to adjust the comorbidities, we used CCI, which is known to predict the 10 year survival of patients who have comorbidities. Second, this study used a claim database, so it was not possible to obtain information on actual clinical information such as laboratory tests, pulmonary function, and respiratory symptoms. We defined COPD and CHD by using a structural definition and there is a possibility of overdiagnosis or misdiagnosis as these diseases were not confirmed by tests such as spirometry or coronary artery angiography. Third, our study showed a comparably higher proportion of women and non-smokers although we targeted COPD patients. This may be due to the limitation that we applied operational definition instead of spirometry results. This proportion could also be explained by our exclusion of patients who did not have any information from the national health examination, which contains smoking status and BMI. It is known that the smoking rate in Korean men is higher than that of Korean women, and smokers tend to have less interest in health examinations compared to non-smokers. Fourth, this study analyzed only the first LAMA, tiotropium. After the release of tiotropium, many new types of LAMA have been released and prescribed. Based on the results of this study alone, it cannot be concluded that LAMAs other than tiotropium have the same effect on CHD as that of tiotropium. Fifth, although the soft mist formulation is known to deliver a higher amount of tiotropium to the lungs than the capsule handihaler formulation, we did not evaluate whether these two formulations of tiotropium differed in their effect on CHD risk. In addition, the cumulative exposure to tiotropium in this study is the prescribed amount and does not reflect actual patient compliance or the amount of drug absorbed into the lungs.

Our study demonstrated that in COPD patients, the higher the cumulative exposure to tiotropium, the higher the risk of CHD. Particularly in those aged 55 years and older and never-smokers, tiotropium administration was associated with an increased risk of CHD. Therefore, careful monitoring is required when prescribing high cumulative doses of tiotropium to the elderly. It is important to reduce the risk of CHD because the occurrence of CHD leads to a poor prognosis of COPD. Considering the risk–benefit ratio of LAMA, additional studies are needed to clarify the optimal dose or duration of LAMA use.

## Supplementary Information

Supplementary Information.

## Data Availability

The NHIS-NSC data that support the findings of this study are available from the NHIS but restrictions apply to the availability of these data, which were used under license for the current study, and so are not publicly available. Data are, however, available from the corresponding author upon reasonable request and with permission of NHIS.
