# Supplementary material for: Effects of tiotropium on the risk of coronary heart disease in patients with COPD: a nationwide cohort study
Source: Sci Rep. 2022 Oct 5;12:16674. doi: 10.1038/s41598-022-21038-1 (PMC9535029; doi:10.1038/s41598-022-21038-1)
Supplement: Supplementary file 1 — Supplementary Information. [file 41598_2022_21038_MOESM1_ESM.docx]

Supplement table 1. Korean Classification of Diseases, 6^th^ revision (KCD-6) codes used for defining COPD and CHD.

| Disease | KCD-6 Code | Description |
| --- | --- | --- |
| Chronic obstructive lung disease (COPD) | J42 | Unspecified chronic bronchitis |
|  | J43 | Emphysema |
|  | J43.1 | Panlobular emphysema |
|  | J43.2 | Centrilobular emphysema |
|  | J43.8 | Other emphysema |
|  | J43.9 | Emphysema, unspecified |
|  | J44 | Other chronic obstructive pulmonary disease |
|  | J44.0 | Chronic obstructive pulmonary disease with acute lower respiratory infection |
|  | J44.1 | Chronic obstructive pulmonary disease with acute exacerbation, unspecified |
|  | J44.8 | Other specified chronic obstructive pulmonary disease |
|  | J44.9 | Chronic obstructive pulmonary disease, unspecified |
| Coronary heart disease (CHD) | I20 | Angina pectoris |
|  | I20.0 | Unstable angina |
|  | I20.1 | Angina pectoris with documented spasm |
|  | I20.8 | Other forms of angina pectoris |
|  | I20.9 | Angina pectoris, unspecified |
|  | I21 | Acute myocardial infarction |
|  | I21.0 | Acute transmural myocardial infarction of anterior wall |
|  | I21.1 | Acute transmural myocardial infarction of inferior wall |
|  | I21.2 | Acute transmural myocardial infarction of other sites |
|  | I21.3 | Acute transmural myocardial infarction of unspecified site |
|  | I21.4 | Acute subendocardial myocardial infarction |
|  | I21.9 | Acute myocardial infarction, unspecified |
|  | I22 | Subsequent myocardial infarction |
|  | I22.0 | Subsequent myocardial infarction of anterior wall |
|  | I22.1 | Subsequent myocardial infarction of inferior wall |
|  | I22.8 | Subsequent myocardial infarction of other sites |
|  | I22.9 | Subsequent myocardial infarction of unspecified site |
|  | I23 | Certain current complications following acute myocardial infarction |
|  | I23.0 | Haemopericardium as current complication following acute myocardial infarction |
|  | I23.1 | Atrial septal defect as current complication following acute myocardial infarction |
|  | I23.2 | Ventricular septal defect as current complication following acute myocardial infarction |
|  | I23.3 | Rupture of cardiac wall without haemopericardium as current complication following acutemyocardial infarction |
|  | I23.4 | Rupture of chordae tendineae as current complication following acute myocardial infarction |
|  | I23.5 | Rupture of papillary muscle as current complication following acute myocardial infarction |
|  | I23.6 | Thrombosis of atrium, auricular appendage, and ventricle as current complicationsfollowing acute myocardial infarction |
|  | I23.8 | Other current complications following acute myocardial infarction |
|  | I24 | Other acute ischaemic heart diseases |
|  | I24.0 | Coronary thrombosis not resulting in myocardial infarction |
|  | I24.1 | Dressler’s syndrome |
|  | I24.8 | Other forms of acute ischaemic heart disease |
|  | I24.9 | Acute ischaemic heart disease, unspecified |
|  | I25 | Chronic ischaemic heart disease |
|  | I25.0 | Atherosclerotic cardiovascular disease, so described |
|  | I25.1 | Atherosclerotic heart disease |
|  | I25.2 | Old myocardial infarction |
|  | I25.3 | Aneurysm of heart |
|  | I25.4 | Coronary artery aneurysm and dissection |
|  | I25.5 | Ischaemic cardiomyopathy |
|  | I25.6 | Silent myocardial ischaemia |
|  | I25.8 | Other forms of chronic ischaemic heart disease |
|  | I25.9 | Chronic ischaemic heart disease, unspecified |

Supplement Table 2. Revascularization procedure codes used for defining coronary heart disease

| Procedure | Procedure Code | Description |
| --- | --- | --- |
| Coronary artery bypass grafting (CABG) | O1641 | Simple Aorta-Coronary Vascular Bypass Operation (Artery), 1 site |
|  | O1642 | Simple Aorta-Coronary Vascular Bypass Operation (Artery), 2 site or more |
|  | O1647 | Complex Aorta-Coronary Vascular Bypass Operation (Artery) |
|  | OA641 | Simple Off Pump CABG Aorta-Coronary Vascular Bypass Operation (Artery), 1 site |
|  | OA642 | Simple Off Pump CABG Aorta-Coronary Vascular Bypass Operation (Artery), 2 site or more |
|  | OA647 | Complex Off Pump CABG Aorta-Coronary Vascular Bypass Operation (Artery) |
| Percutaneous coronary intervention (PCI) | M6551 | Percutaneous Transluminal Coronary Angioplasty, single vessel |
|  | M6552 | Percutaneous Transluminal Coronary Angioplasty, additional vessel |
|  | M6561 | Percutaneous Trans-catheter Placement of Intracoronary Stent, single vessel |
|  | M6562 | Percutaneous Trans-catheter Placement of Intracoronary Stent, additional vessel |
|  | M6563 | Percutaneous Trans-catheter Placement of Intracoronary Stent with angioplasty or atherectomy, single vessel |
|  | M6564 | Percutaneous Trans-catheter Placement of Intracoronary Stent with angioplasty or atherectomy, additional vessel |
|  | M6571 | Percutaneous Transluminal Coronary Atherectomy, single vessel |
|  | M6572 | Percutaneous Transluminal Coronary Atherectomy, additional vessel |

Supplementary Table 3. Association between tiotropium administration and the risk of coronary heart disease in COPD patients including interaction term in the model^a^

|  | No. of CHD cases/ Total No. of subgroup patients | Adjusted hazard ratio  (95% CI) | p value |
| --- | --- | --- | --- |
| Model 1^b^ | 1,074/5,787 | 1.23 (1.00-1.51) | 0.052 |
| Model 2^c^ |  | 1.07 (0.53-2.18) | 0.848 |
| Model 3^d^ |  | 2.40 (1.19-4.87) | 0.015^f^ |
| Model 4^e^ |  | 1.16 (0.71-1.89) | 0.547 |
| COPD, chronic obstructive; LAMA, long-acting muscarinic antagonists; CI, confidence interval  ^a^ LAMA usage is analyzed as time-dependent covariates and converted to mg.  ^b^ Model 1 was adjusted for age, sex, body mass index, household income level, Charlson comorbidity index, and smoking status,  ^c^ Model 2 was adjusted for Model 1 + interaction term between LAMA and age group (Age $\geq$ 55, Age < 55).  ^d^ Model 3 was adjusted for Model 1 + interaction term between LAMA and sex.  ^e^ Model 4 was adjusted for Model 1 + interaction term between LAMA and smoking status  ^f^ Interaction term had a p-value < 0.1 | | | |

Supplementary Table 4. Association between cumulative dose of tiotropium exposure and the risk of coronary heart disease in COPD patients including interaction term in the model^a^

|  | No. of CHD cases/ Total No. of subgroup patients | Adjusted hazard ratio  (95% CI) | p value |
| --- | --- | --- | --- |
| Model 1^b^ | 1,074/5,787 | 1.01 (1.002-1.022) | 0.020 |
| Model 2^c^ |  | 1.00 (0.95-1.05) | 0.996 |
| Model 3^d^ |  | 1.01 (0.97-1.04) | 0.642 |
| Model 4^e^ |  | 1.05 (1.02-1.07) | <0.01^f^ |
| COPD, chronic obstructive; LAMA, long-acting muscarinic antagonists; CI, confidence interval  ^a^ LAMA usage is analyzed as time-dependent covariates and converted to mg.  ^b^ Model 1 was adjusted for age, sex, body mass index, household income level, Charlson comorbidity index, and smoking status,  ^c^ Model 2 was adjusted for Model 1 + interaction term between LAMA and age group (Age $\geq$ 55, Age < 55).  ^d^ Model 3 was adjusted for Model 1 + interaction term between LAMA and sex.  ^e^ Model 4 was adjusted for Model 1 + interaction term between LAMA and smoking status  ^f^ Interaction term had a p-value < 0.1 | | | |

Supplementary Table 5. Association between tiotropium administration and the risk of coronary heart disease in COPD patients aged 40 and over^a^

| Subgroup | Crude hazard ratio  (95% CI) | P | Adjusted hazard ratio  (95% CI) | P |
| --- | --- | --- | --- | --- |
| Total^b^ | **1.48 (1.21-1.81)** | **<0.001** | 1.23 (1.00-1.51) | 0.054 |
| Adults ≥ 55 years ^b^ | **1.41 (1.14-1.74)** | **0.002** | **1.24 (1.003-1.54)** | **0.047** |
| Males < 55 years ^c^ | 1.82 (0.82-4.02) | 0.14 | 1.67 (0.74-3.75) | 0.22 |
| Females < 55 years ^c^ | 0.35 (0.05-2.51) | 0.30 | 0.41 (0.06-3.01) | 0.38 |
| Never smoker ^d^ | **1.52 (1.13-2.05)** | **0.01** | 1.15 (0.85-1.55) | 0.38 |
| Former smoker ^d^ | 1.37 (0.90-2.07) | 0.14 | 1.30 (0.85-1.97) | 0.23 |
| Current smoker ^d^ | **1.54 (1.06-2.25)** | **0.03** | 1.33 (0.91-1.95) | 0.15 |
| ^a^ LAMA usage is analyzed as time-dependent covariates  ^b^ Adjusted HRs were adjusted for age, sex, body mass index, household income level, Charlson comorbidity index, and smoking status  ^c^ Adjusted HRs were adjusted for age, body mass index, household income level, Charlson comorbidity index, and smoking status  ^d^ Adjusted HRs were adjusted for age, sex, body mass index, household income level, and Charlson comorbidity index | | | | |

Supplementary Table 6. Association between cumulative dose of tiotropium exposure and the risk of coronary heart disease in COPD patients aged 40 and over^a^

| Subgroup | Crude hazard ratio  (95% CI) | P | Adjusted hazard ratio  (95% CI) | P |
| --- | --- | --- | --- | --- |
| Total^b^ | **1.02 (1.01-1.03)** | **<0.001** | **1.01 (1.002-1.02)** | **0.020** |
| Adults ≥ 55 years ^b^ | **1.02 (1.01-1.03)** | **0.002** | **1.01 (1.002-1.022)** | **0.015** |
| Males < 55 years ^c^ | 1.00 (0.93-1.07) | 0.98 | 1.00 (0.93-1.08) | 0.94 |
| Females < 55 years ^c^ | 1.03 (0.96-1.11) | 0.42 | 0.98 (0.91-1.06) | 0.64 |
| Never smoker ^d^ | **1.04 (1.02-1.05)** | **<0.001** | **1.03 (1.02-1.04)** | **<0.001** |
| Former smoker ^d^ | 0.99 (0.96-1.02) | 0.62 | 1.00 (0.96-1.03) | 0.77 |
| Current smoker ^d^ | 1.01 (0.98-1.04) | 0.53 | 1.00 (0.97-1.02) | 0.80 |
| ^a^ LAMA usage is analyzed as time-dependent covariates and converted to mg.  ^b^ Adjusted HRs were adjusted for age, sex, body mass index, household income level, Charlson comorbidity index, and smoking status  ^c^ Adjusted HRs were adjusted for age, body mass index, household income level, Charlson comorbidity index, and smoking status  ^d^ Adjusted HRs were adjusted for age, sex, body mass index, household income level, and Charlson comorbidity index | | | | |
